# Process Evaluation of a Structured Method for Systematic and Integrated Occupational Safety and Health and Patient Safety Management Systems (SIOHPS): Protocol for a Convergent Parallel Mixed Methods Study

**DOI:** 10.2196/89185

**Published:** 2026-01-27

**Authors:** Camilla Göras, Ann-Sofie Ersson, Therese Hellman, Petronella Bjurling-Sjöberg, Gunnar Bergström, Robert Sarkadi Kristiansson, Malin Lohela-Karlsson

**Affiliations:** 1 Department of Caring Sciences Faculty of Health and Occupational Sciences University of Gävle Gävle Sweden; 2 Department of Caring Sciences School of Health and Welfare Dalarna University Falun Sweden; 3 Centre for Clinical Research Västmanland Uppsala University Västerås, Uppsala Sweden; 4 Department of Public Health and Caring Sciences Uppsala University Uppsala Sweden; 5 Department of Medical Sciences Uppsala University Uppsala, Uppsala Sweden; 6 Primary Care and Health Region Uppsala Uppsala Sweden; 7 Centre for Clinical Research Sörmland Uppsala University Eskilstuna Sweden; 8 Unit of Intervention and Implementation Research for Worker Health Karolinska Institutet Stockholm Sweden; 9 Department of Occupational Health, Psychology and Sports Sciences Faculty of Health and Occupational Studies University of Gävle Gävle Sweden; 10 School of Health, Care and Social Welfare Mälardalen University Västerås Sweden

**Keywords:** complex intervention, health care, mixed methods, occupational safety and health, patient safety, process evaluation, study protocol

## Abstract

**Background:**

Health care systems are increasingly challenged by demographic shifts, rising chronic illnesses, human resource constraints, and growing efficiency demands. Improving both occupational safety and health (OSH) and patient safety (PS) management has been identified as a pivotal strategy to address these challenges. However, there is a paucity of evidence-based methods that support systematic and integrated OSH and PS. In response, the Systematic and Integrated Occupational Safety and Health and Patient Safety Management Systems (SIOHPS) intervention was developed, guided by the Safer Culture Framework and the Medical Research Council (MRC) framework for complex interventions.

**Objective:**

This paper outlines the study protocol for a process evaluation embedded in the SIOHPS trial.

**Methods:**

The process evaluation will use a convergent parallel mixed methods design. The SIOHPS trial is conducted in 13 Swedish hospital settings. Guided by the Consolidated Framework for Implementation Research (CFIR), quantitative and qualitative data will be collected before, during, and after the trial through questionnaires, telephone interviews, focus group interviews (FGIs), observations, the SIOHPS digital tool, and other relevant documentation. In line with the convergent parallel mixed methods study design, the quantitative and qualitative data will be analyzed in a stepwise manner, initially independently from each other, followed by iterative triangulation.

**Results:**

Funding began in January 2023. The development phase was completed in early 2024, and the evaluation phase started in June 2024, with completion planned in early February 2026. Quantitative data collection for two of three clusters (baseline, 4- and 8-month follow-up) is complete, and data cleaning is underway. All qualitative data collected to date have been transcribed. Final data collection for cluster III, including the 8-month survey and FGIs, is scheduled for the end of January and early February 2026. Data analysis will begin in early 2026, with results to be disseminated through publications and conference presentations later in 2026.

**Conclusions:**

The process evaluation will integrate quantitative and qualitative data sources to elucidate the mechanisms through which the SIOHPS intervention influences safety culture, health care worker (HCW) health, PS, and quality of care. This comprehensive approach unpacks the “black box” of the implementation process, which will provide an in-depth nuanced picture of the intervention’s effectiveness and valuable insights into scalability and transferability across diverse health care contexts.

**Trial Registration:**

ClinicalTrials.gov NCT06398860; https://clinicaltrials.gov/study/NCT06398860

**International Registered Report Identifier (IRRID):**

DERR1-10.2196/89185

## Introduction

Health care systems are increasingly challenged by demographic shifts, rising chronic illness, human resource constraints, and growing efficiency demands [1]. These challenges complicate the delivery of high-quality and safe care [2], place significant strain on the work environment of health care workers (HCWs), and threaten the sustainability of health care. Occupational safety and health (OSH) and patient safety (PS) are closely intertwined fields [3]. The COVID-19 pandemic underscored this interdependence, emphasizing that protecting HCW well-being is essential to maintaining a resilient health care system [4].

Integrated OSH and PS management, to simultaneously prevent adverse outcomes in both fields, has been raised as having potential for addressing the challenges in a resource-effective manner [4,5]. However, evidence-based methods integrating OSH and PS are lacking (M Lohela-Karlsson, Associate Professor, unpublished data, January 2026), highlighting the need for the development and evaluation of such methods. In response, a theoretically grounded project was launched to develop and evaluate a structured method for OSH and PS management, called Systematic and Integrated Occupational Safety and Health and Patient Safety Management Systems (SIOHPS). To evaluate the SIOHPS intervention, a large-scale multidisciplinary trial has been performed [6].

From a theoretical perspective [7], effective integration of OSH and PS requires addressing organizational factors that support a culture of safety across multiple system levels. This complexity needs to be considered in intervention development and evaluation, preferably codesigning it with key stakeholders and addressing the needs of different system levels. However, the efficiency of complex interventions, defined by multiple interacting components, diverse outcomes, and system-level impact [8], depends not only on the intervention itself, but also on the implementation process [9,10] and sustained enactment [11].

The viability of an intervention depends on implementation outcomes, that is, stakeholders’ satisfaction with the intervention (acceptability); its fit and relevance to the context, problem, and users (appropriateness); the extent to which it can be successfully deployed (feasibility); and the financial impact and resources required for the implementation effort (implementation cost) [8]. Additionally, the extent to which an intervention is implemented as intended (fidelity) by its developers is critical for internal validity and for understanding intervention effectiveness [12]. Context-sensitive adaptations of an intervention’s design or delivery (adaptations) may be needed, while preferably preserving its core components [13]. Once viewed as a threat to fidelity, adaptations are currently considered vital for aligning complex interventions with real-world contexts and enhancing implementation outcomes [14]. Similarly, the extent to which an implemented intervention is sustained over time (sustainability) has been seen as a final implementation outcome [15], while being contemporary viewed as a dynamic, adaptive process that ensures continued use of an intervention in response to evolving needs [10]. Implementation outcomes are also shaped by the process through which interventions produce change, that is, enabling factors, enacting behaviors, and eventual unexpected pathways and consequences (mechanisms of impact) [7]. Hence, facilitators and barriers in the organizational, cultural, and structural environment in which an intervention is implemented (contextual conditions) [16,17] can provide valuable insights into intervention effectiveness [18].

Implementation processes of complex interventions are often referred to as the “black box,” insinuating a lack of transparency that undermines reliable interpretation of outcomes and eventual scalability across health care settings. Additionally, despite growing interest, few studies address sustained interventions, probably due to varied definitions of sustainability and challenges in adapting interventions across contexts [19]. Addressing questions beyond the scope of effectiveness studies, process evaluations unpack the “black box” of the implementation process, with the purpose to understand how and why interventions succeed or fail. Such understanding can be used to assess whether interventions are viable and what adaptations and implementation strategies might be suitable in eventual future implementation across diverse contexts [18]. This paper outlines the study protocol for a process evaluation embedded in the SIOHPS trial [6].

The overall aim of this process evaluation is to understand how the SIOHPS intervention is delivered and adhered to in practice, how context influences implementation, and the mechanisms through which it produces change.

The specific aims of the process evaluation of the SIOHPS intervention are to:

- Describe the content, frequency and dose, duration, and coverage (fidelity), as well as adaptations for contextual fit.
- Explore the contextual factors that facilitate or hinder the implementation of the intervention.
- Describe and compare short- and long-term outcomes between settings in relation to the implementation process.
- Identify variables (mechanisms of impact) that may influence or contribute to the development of the intervention outputs.

## Methods

### Study Design

This study was registered at ClinicalTrials.gov (NCT06398860). The process evaluation follows the Medical Research Council (MRC) framework for complex interventions [18]. A convergent parallel mixed methods design [20] is used within a hybrid type 1 effectiveness-implementation framework.

**Figure 1:**
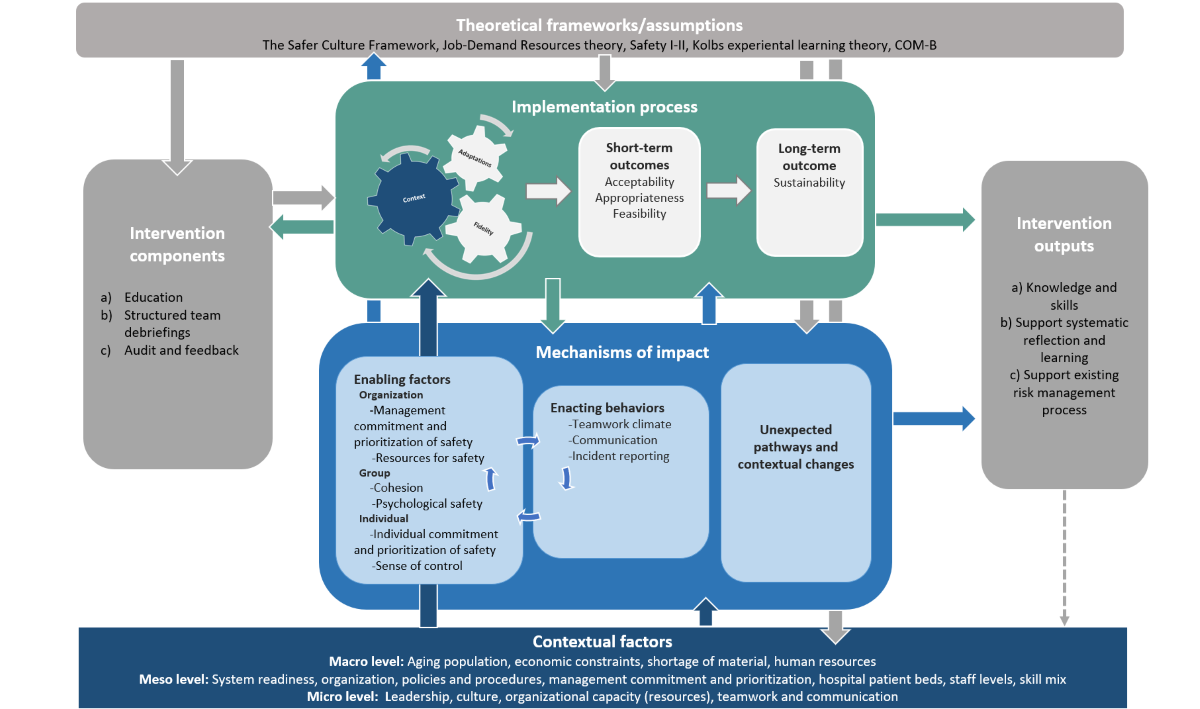
Logic model illustrating the interrelationship between the process evaluation components: contextual factors; the implementation process and mechanisms of impact (coloured boxes); and the theoretical foundation, core components of the intervention, and expected intervention outputs (grey boxes). COM-B: capability, opportunity, motivation, and behavior.

The following section briefly describes the overall design and setting of the SIOHPS project. Next, the intervention (the SIOHPS method) and its underlying theoretical assumptions are provided, visualized in a logic model (Figure 1). Thereafter, participants, the procedure, and the process evaluation are outlined, including domains of the evaluation and the theoretical rationale for studying them. Finally, the planned analysis is described.

### The SIOHPS Project

The SIOHPS project evaluates whether a structured, integrated approach to managing OSH and PS can improve HCW health and quality of care. It follows the MRC framework for complex interventions [8], emphasizing stakeholder codesign and contextual adaptation. The project is led by a core team consisting of the principal investigator (PI); two regional project managers, responsible for coordinating units in respective regions; and an administration assistant. The core team members bring complementary research and clinical expertise from health care fields, such as OSH management, occupational health services, PS, and PS culture. An advisory board (with clinical and academic background and expertise, including OSH, PS, and safety culture; intervention and implementation research; and roles such as chief medical officer, PS coordinator, and statistician) provides ongoing guidance.

The project comprised a development phase (2023-2024); it also comprises an evaluation phase. During the development phase, the program theory, intervention components, and evaluation design were refined in accordance with O’Cathain et al [21]. This work was coproduced with key stakeholders, including managers, HCWs involved in PS tasks, safety representatives, and central organizational functions. The development phase will be reported in a separate paper.

The evaluation uses a large-scale, multidisciplinary hybrid type 1 effectiveness-implementation framework [22], with the primary focus on effectiveness and cost-effectiveness and the secondary focus on the implementation process. The effectiveness study uses a pragmatic stepped-wedge cluster-controlled trial across two Swedish regions, involving 13 hospital units with round-the-clock care. Units transition from control to intervention in three steps, with at least 4 units per step and ≥360 HCWs. The sample size was determined to ensure sufficient unit-level representation, acknowledging variation in HCW numbers across units. All participating units had to provide round-the-clock care. The study protocol for the trial is outlined in a separate paper [6], while further details of the process evaluation are outlined later. Evaluation of effectiveness and process will be conducted by two separate but collaborative teams, led by the PI and one of the regional project managers, respectively. Integrating the teams supports coherent data collection, prevents duplication of effort, minimizes participant burden, and enables meaningful integration between implementation data and process outcomes [18].

The PI holds overall responsibility for ensuring the evaluation of established study protocols. The evaluation design, including the development of data collection instruments, was carried out in close collaboration with the advisory board during the development phase. The advisory board will continue to be consulted and receive regular updates, providing feedback on interim findings and overall implementation.

### The SIOHPS Intervention

The developed intervention (ie, the SIOHPS method) consists of three core components: (1) *targeted education*, delivered in live format or in online format provided by the research team, to build foundational knowledge and practical skills to adhere to the SIOHPS method; (2) *team debriefings* (also known as end-of-shift huddles or after-action reviews), which HCWs are encouraged to perform after every shift; and (3) monthly *audit and feedback* by management in collaboration with the support functions based on data from team debriefings (Figure 1). The components target the needs of different system levels, involving management at both organizational and group levels, support functions (including those involved in PS work and the safety representatives of the work environment), and HCWs. The method encourages an integrated OSH and PS perspective when identifying patterns and root causes and taking measures, supported by a novel digital tool to be used in team debriefings, audit, and feedback.

### Theoretical Assumptions

The SIOHPS intervention and its process evaluation is theoretically grounded in the principles of safety culture theory [7] and complemented with four additional theoretical foundations: job demand-resources theory [23], the Safety I-II approach [24], Kolb’s experiential learning theory [25], and the COM-B (capability, opportunity, motivation, and behavior) model for behavior change [26]. To help clarify causal assumptions, a logic model was developed by the core team based on the relevant literature and in collaboration with the advisory board (Figure 1).

Briefly, the logic model visualizes that to evaluate the *implementation* and sustainability of SIOHPS, it is crucial to consider fidelity, adaptation, and context, as these domains interact in dynamic ways. The fidelity-adaptation dilemma [14,16,27] highlights the tension between preserving the core components of an intervention and adapting it to the local context. Striking this balance is crucial for sustainability and effectiveness, shaped by contextual factors across system levels (micro, meso, and macro) [16]. The core components of the *intervention* are designed to address the theoretical *mechanisms of impact* (ie, enabling factors and enacting behaviors) that, based on the Safer Culture Framework, influence the safety culture in the workplace [7]. Although the enabling factors do not constitute an integral component of SIOHPS or the chain of effects, they are nevertheless considered a contextual factor that possesses the capacity to exert influence on the outcome of the intervention. Additionally, unexpected pathways and consequences, due to contextual conditions and changes, affect both short- and long-term process outcomes and intervention output.

In the short term, the expected intervention output includes enhanced HCW participation and learning, alongside improved working conditions (eg, job demand and resources) and improved safety culture. These improvements are anticipated to positively affect work engagement [28], HCW health [29,30], productivity and PS [31-33], and perceived quality of care [31], contributing to reduced sick leave and improved health care quality [33,34]. The intermediate- and long-term effects are hypothesized to be mediated by improved safety culture and psychological safety [33]. However, both short- and long-term outcomes are dependent on the implementation process. Depending on whether participants adhere to the intervention (fidelity adaptations), effects are achieved on staff-related outcomes, patient-related outcomes, and safety culture (OSH culture and PS culture). Adherence to the intervention can be explained by the COM-B model [26].

### Participants and Procedure

**Figure 2:**
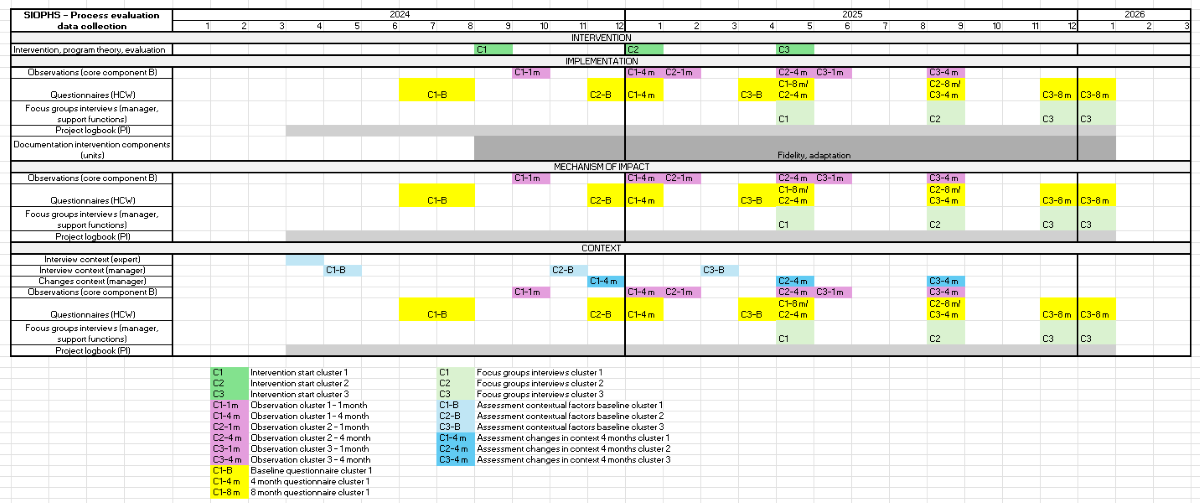
Gantt chart illustrating data collection during the process evaluation phase, visualized in relation to the process evaluation components in accordance with the logic model. The boxes represent the type of data collected, the timing, and the month from intervention start when each data collection activity was conducted. C: cluster (C1-C3); HCW: health care worker; m: month (1=1 month, 4=4 months, 8=8 months); Pl: project logbook; SIOHPS: Systematic and Integrated Occupational Safety and Health and Patient Safety Management Systems.

Participants will include nursing assistants, registered nurses, physicians, managers, paramedical services, and support functions, together with internal facilitators. Stakeholders at the meso level, those functions involved in OSH and PS, will also contribute as informants. All HCWs will be encouraged to take part in the intervention, but to participate in the study, they must work at least 50% full-time at the unit. Data will be collected from various participants and stakeholders throughout the project period using a range of quantitative and qualitative methods. The sample size has been determined pragmatically for various parts of the project. The number of units included and HCWs was based on a power calculation for the intervention effect. Each unit was assumed to include an average of 30 HCWs. Based on the sample size calculation, at least 12 units and 360 HCWs were required to be included in the study to achieve sufficient statistical power for further analyses. The estimate was sufficiently conservative to detect the lowest expected intervention effect. The sample size was considered a minimum requirement, with primary emphasis placed on achieving the estimated number of units, as the number of staff members varied across units. A dropout rate of less than 20% for participating units and an attendance rate of at least 70% of all HCWs have been assumed in this study. For further details, please see the effectiveness study protocol [6]. The data collection process is illustrated in a Gantt chart (Figure 2). Overviews of the explored process domains, data collection methods, and their operationalization in relation to the three core components of SIOHPS are presented in Tables 1-4 for the *implementation process* and Tables 5 and 6 for the *mechanisms of impact*.

Survey data from HCWs will be collected in parallel with the effectiveness evaluation and comprise both quantitative measures (eg, Likert-scale items) and qualitative data from open-ended questions (Tables 1-6). All HCWs who fulfill the inclusion criteria will be invited to answer the questionnaires at each of the three measurement points (baseline, 4 months, and 8 months). Additional quantitative data will be collected from the SIOHPS digital tool. Qualitative data will be collected through individual telephone interviews with managers at each health care unit at the beginning of the study (n=13) and after 4 months (n=13). Focus group interviews (FGIs) with managers, support functions, and internal facilitators will be conducted at each health care unit after 8 months (n=13).

The performance of the SIOHPS method during team debriefings will be examined through nonparticipating observations conducted twice at each unit, after 1 and 4 months, respectively (n=at least 1 observation per data collection, yielding a minimum of 26 observations in total). The observations will be conducted by a single researcher and will not audio- or video-recorded in order to minimize potential inhibitory effects on team behavior. The observations will be documented contemporaneously in a template, and reflexive field notes will be documented after each observation. The observation protocol comprises two parts: intervention fidelity and psychological safety. The intervention fidelity part will be based on the SIOHPS method used during team debriefings. The psychological safety part will be based on O’Donovan et al’s work [35], with adjustments to fit debriefings under a short duration with possible interruptions. The observer will be one of the researchers (author ASE), who is well informed about the intervention and the study. Additionally, the observer will be trained in pilot observations, in which the observer and the PI independently will rate the same team debriefings. These will be discussed to ensure consistency in interpretation. The protocol will be refined accordingly, followed by additional pilot observations to further train the observer and to ensure that the protocol functions according to its purpose.

Documentation in logbooks will occur iteratively during the different phases, as described in the MRC framework [18]. Additionally, qualitative data will be gathered through face-to-face interactions, telephone calls, video conferences and informal observations, and continuous process notes documented in connection with every contact with participating units.

All data will be stored pseudonymized, with only the date and time of the interviews documented, and will be analyzed solely at the group level.

**Table 1:** Overview of the “fidelity” process domain, data collection, and operationalization in relation to the three core components for exploration of the SIOHPS^a^ implementation.

Dimension, core component, and data sources		Data collection methods (number of planned data collections, where relevant)	Operationalization	Data type
**Content and targeted education**				
	First-line managers and support functions	FGIs^b^ (n=13)	Experience of the targeted education	Qualitative
**Content and team debriefings**				
	HCWs^c^	Direct observations (≥26)	Adherence to the protocol	Quantitative
	HCWs	Direct observations (≥26)	Interruptions and the causes of observed interruptions	Quantitative, qualitative
	First-line managers and support functions	FGIs (n=13)	Experience of structured team debriefings	Qualitative
**Content, and audit and feedback**				
	First-line managers and support functions	FGIs (n=13)	Experience of audit and feedback	Qualitative
**Duration and team debriefings**				
	HCWs	Direct observations (≥26)	Duration of structured team debriefings	Quantitative
**Duration, and audit and feedback**				
	First-line managers	Documentation and FGIs (n=13)	Experience of audit and feedback	Qualitative
	HCWs	Questionnaires (n=2)	Perception of regular feedback	Quantitative
**Frequency and dose, and targeted education**				
	HCWs	Questionnaires (n=2)	Participation in targeted education and in what format	Quantitative
	First-line managers and support functions	Documentation and FGIs	Participation in targeted education and in what format	Quantitative, qualitative
**Frequency and dose, and team debriefings**				
	SIOHPS digital tool	All HCWs	Number of reported estimates, including distribution by work shift	Quantitative
	HCWs	Questionnaires (n=2)	Participation in structured team debriefings in the past 4 months	Quantitative
**Frequency and dose, and audit and feedback**				
	First-line managers and support functions	FGIs (n=13)	Experience of conducted audit and feedback	Qualitative
	HCWs	Questionnaires (n=2)	Perception of regular feedback on reported data in the SIOHPS digital tool	Quantitative
**Coverage (reach) and targeted education**				
	First-line managers and support functions	Documentation/logbook	Participation in targeted education and in what format	Qualitative
**Coverage (reach) and team debriefings**				
	First-line managers and support functions	FGIs (n=13)	Presence during team debriefings	Qualitative
	HCWs	Direct observations (≥26) and questionnaires (n=2)	Participating professionals during structured team debriefings	Quantitative, qualitative
**Coverage (reach), and audit and feedback**				
	First-line managers and support functions	FGIs	Providing audit and feedback	Qualitative
	HCWs	Questionnaires (n=2)	Perception of regular feedback on reported data in the SIOHPS digital tool	Quantitative

^a^SIOHPS: Systematic and Integrated Occupational Safety and Health and Patient Safety Management Systems.

^b^FGI: focus group interview.

^c^HCW: health care worker.

**Table 2:** Overview of the “adaptations” process domain, data collection, and operationalization in relation to the three core components for exploration of the SIOHPS^a^ implementation.

Core components and data source		Data collection methods (number of planned data collections, where relevant)	Operationalization	Data type
**Targeted education**				
	First-line managers	Documentation in a logbook	Contextual adaptations of education sessions	Qualitative
**Targeted education and team debriefings**				
	HCWs^b^	Direct observations with memos (≥26)	Adaptations from the original protocol and as intended by developers	Qualitative
	HCW	A follow-up question postobservation (≥26)	Experiences of adaptation needs, the cause, and adaptations implemented	Qualitative

^a^SIOHPS: Systematic and Integrated Occupational Safety and Health and Patient Safety Management Systems.

^b^HCW: health care worker.

**Table 3:** Overview of the “context” process domain, data collection, and operationalization in relation to the three core components for exploration of the SIOHPS^a^ implementation.

Core components and data sources			Data collection methods (number of planned data collections, where relevant)		Operationalization		Data type
**Targeted education, team debriefings, and audit and feedback**							
	Central support functions working within the area of PS^b^ or the work environment and first-line managers	Telephone interviews (n=13+4)		Mapping of factors at micro and meso levels with a potential impact on the conditions for systematic quality and safety work related to the work environment and PS		Quantitative, qualitative	
	First-line managers	Telephone interviews (n=13)		Experience of organizational/contextual changes		Qualitative	
	First-line managers and support functions	FGIs^c^ (n=13)		Experience of barriers and facilitators		Qualitative	
	Logbook	Documentation		Contextual changes over time		Qualitative	

^a^SIOHPS: Systematic and Integrated Occupational Safety and Health and Patient Safety Management Systems.

^b^PS: patient safety.

^c^FGI: focus group interview.

**Table 4:** Overview of the “short- and long-term outcomes” process domain, data collection, and operationalization in relation to the three core components for exploration of the SIOHPS^a^ implementation.

Dimension, core components, and data sources		Data collection methods (number of planned data collections, where relevant)	Operationalization	Data type
**Acceptability, appropriateness, and feasibility (short term); team debriefings**				
	HCWs^b^	Questionnaires (n=2)	Perception of the SIOHPS digital tool	Quantitative
**Acceptability, appropriateness, and feasibility (short term); targeted education, team debriefings, and audit and feedback**				
	First-line managers and support functions	FGIs^c^ (n=13)	Experience of the SIOHPS method	Qualitative
**Sustainability (long term); team debriefings**				
	HCWs	Questionnaires (n=2)	Perception of motivational factors involvement, communication, psychological safety, and consequences	Quantitative
	HCWs	Questionnaires (n=2)	Perception of intervention complexity, compatibility, and advantages of the method	Quantitative
**Sustainability (long term); audit and feedback**				
	First-line managers and support functions	FGIs (n=13)	Experiences of intervention complexity and advantages of the method	Qualitative
**Sustainability (long term); targeted education, team debriefings, and audit and feedback**				
	HCWs	Questionnaires (n=2)	Perception of management commitment	Quantitative
	First-line managers	FGIs (n=13)	Experience of support from head of department	Qualitative
	First-line managers and support functions	FGIs (n=13)	Experience of preconditions and goals	Qualitative
**Sustainability (long term); team debriefings, and audit and feedback**				
	First-line managers and support functions	FGIs (n=13)	Experience of internal and external support	Qualitative

^a^SIOHPS: Systematic and Integrated Occupational Safety and Health and Patient Safety Management Systems.

^b^HCW: health care worker.

^c^FGI: focus group interview.

**Table 5:** Overview of the “enabling factors” process domain, data collection, and operationalization in relation to the three core components for exploration of the mechanisms of impact.

System level and factors		Data source	Data collection methods (number of planned data collections, where relevant)	Operationalization	Data type
**Organizational level**					
	Management commitment and prioritization of safety	First-line managers and support functions	FGIs^a^ (n=13)	Experiences of SIOHPS^b^	Qualitative
	Resources for safety	First-line managers and support functions	FGIs (n=13)	Experiences of SIOHPS	Qualitative
**Group level**					
	Cohesion	HCWs^c^	Questionnaire	Perceptions of communication and participation	Quantitative
	Psychological safety	HCWs	Questionnaire	Perceptions of psychological safety	Quantitative
**Individual level**					
	Individual commitment and prioritization of safety	HCWs	Questionnaire	Perceptions of work engagement	Quantitative
	Sense of control	HCWs	Questionnaire	Perceptions of resources	Quantitative

^a^FGI: focus group interview.

^b^SIOHPS: Systematic and Integrated Occupational Safety and Health and Patient Safety Management Systems.

^c^HCW: health care worker.

**Table 6:** Overview of the “enacting factors” process domain, data collection, and operationalization in relation to the three core components for exploration of the mechanisms of impact.

Factors		Data source	Data collection methods (number of planned data collections, where relevant)	Operationalization	Data type
Teamwork climate		HCWs^a^	Questionnaire	Perceptions of quality of collaboration between team members	Quantitative
Communication		HCWs	Questionnaire	Perceptions of communication	Quantitative
Incident reporting		Incident-reporting system	—^b^	Frequency of reported incidents	Quantitative
**Unexpected pathways and consequences**					
	Contextual conditions and changes	First-line managers	Telephone interviews (n=13)	Experienced contextual conditions and changes	Qualitative
	Contextual conditions and changes	First-line managers	FGIs^c^ (n=13)	Barriers and facilitators	Qualitative

^a^HCW: health care worker.

^b^Not applicable.

^c^FGI: focus group interview.

### Process Evaluation

The process evaluation will explore the implementation process, including fidelity, adaptations, context and process outcomes (short and long term), and mechanisms of impact [18], with theoretical assumptions based on the logic model (Figure 1). An overview of explored process domains, data collection, and operationalization in relation to the three core components of SIOHPS is presented in Tables 1-6.

#### Implementation Process

The implementation process describes how the intervention is delivered and adhered to in practice [18]. During this process, particular attention will be given to *intervention fidelity*, *adaptations*, and *contextual conditions*, as these factors are expected to influence both *short-* and *long-term outcomes*. Short-term outcomes, including acceptability, appropriateness, and feasibility, are primarily assumed to impact the intervention output and also sustainability of the intervention.

#### Fidelity

Given that SIOHPS is evaluated using an effectiveness-implementation hybrid design, fidelity will be assessed primarily as intervention fidelity, including fidelity of delivery and participant adherence. Fidelity of delivery refers to the interventionists’ actions and reflects the quality, consistency, and alignment of delivered activities in relation to the intervention protocol. Participant adherence captures the extent to which participants engage with the intervention as intended [36]. This part will be explored across four dimensions (*content*, *coverage*, *frequency* and *dose*, and *duration* [10]) related to the three core components of SIOHPS using multiple data sources.

Content will be explored through participants’ experiences of education, information, and support provided for systematic management, complemented by observations of adherence to protocol, interruptions, and the causes of interruptions during team debriefings. Experience of the content in team debriefings and the audit and feedback process will also be examined.

The duration of team debriefings will be observed, and the approximate time spent preparing presentation materials for audit and feedback sessions during workplace meetings will be obtained from the FGIs.

Frequency and dose will be assessed through participation in the targeted education, including the delivery format. Additional information includes the frequency and distribution of team debriefings across work shifts and patterns of participation over time. The audit and feedback component will be examined by assessing the regularity of feedback provided through the SIOHPS digital tool and through routine workplace meetings or other communication channels.

Coverage will be evaluated by documenting participation in education (live or online), presence during team debriefings, and the professional groups represented. Materials used when presenting audit and feedback results in workplace meetings will be collected as part of the documentation. Participants will also be surveyed regarding the regularity of feedback received based on reports in the SIOHPS digital tool.

#### Adaptations

To explore adaptations, information on participation in targeted education and the format will be collected as these are essential for contextual fit. In addition, experiences regarding the need for adaptations, their underlying causes, and the specific adaptations implemented will also be explored.

#### Context

The context domain will be guided by the Consolidated Framework for Implementation Research (CFIR) [37]. Prior to the start of the intervention, a comprehensive mapping of contextual factors in participating regions and units will be conducted through telephone interviews. A follow-up after 4 months will be performed to identify any organizational changes that could influence the implementation of SIOHPS. In addition, barriers and facilitators will be explored following completion of the intervention. Contextual changes during the study period will be continuously documented in the project logbook.

#### Short- and Long-Term Outcomes

During the implementation process, fidelity, adaptation, and contextual factors are expected to influence short-term outcomes, such as *acceptability*, *appropriateness*, and *feasibility*, which, in turn, are expected to mediate long-term outcomes, such as *sustainability*.

Acceptability, appropriateness, and feasibility of the SIOHPS digital tool will be assessed by examining perceptions of its complexity, compatibility, and perceived advantages. Experiences of the SIOHPS method as a whole will also be explored.

To examine long-term sustainability, motivational factors, such as involvement, will be assessed. Perceptions of management commitment will also be collected, and experiences of organizational support, prerequisites, and goals will be explored.

### Mechanisms of Impact

Mechanisms of impact refer to the processes through which interventions produce change. For SIOHPS, these mechanisms are explored from a theoretical perspective, with *enabling factors* from the Safer Culture Framework. The Safer Culture Framework [7] and the COM-B model [26] informed the operationalization of the mechanisms of impact and guided data collection through questionnaires, telephone interviews, and FGIs (Tables 5 and 6).

At the *organizational level*, management commitment and prioritization of safety and investment in safety competencies are expected to foster an environment conducive to effective implementation and sustained impact by enhancing fidelity, promoting safety culture, motivating HCWs, and enabling capacity building. On a *group level,* cohesion and psychological safety facilitate open communication, engagement, and collective learning through trust, key processes for cultural change, and long-term sustainability. At the *individual level*, commitment, prioritization of safety, and perceived control are assumed to be drivers of sustained engagement.

Together, these enabling factors influence *enacting behaviors*, such as communication, teamwork, incident reporting, and ownership of safety practices, thereby activating mechanisms for lasting improvements in OSH culture [7]. SIOHPS is designed to reinforce these processes by promoting effective communication and regular incident reporting.

Contextual factors and organizational changes may also generate unanticipated pathways influencing short- and long-term outcomes, reflecting the dynamic interplay between external demands, internal dynamics, and organizational structures.

### Data Management and Analysis

In line with the MRC framework [18], qualitative findings will be integrated with quantitative measures of process variables and short- and long-term outcomes, particularly where these are expected to influence the SIOHPS intervention’s effectiveness and functionality. All analyses will be explorative and framed by the underlying logic model (Figure 1). Aligned with a developmental evaluation approach, the analysis will remain open to emergent findings and local contextual adaptations, supported by continuous learning [38]. This will enable the study to capture how the intervention functions in practice and to identify refinements for future large-scale implementation [8,39].

#### Quantitative Data

For all quantitative data (Tables 1-6), analyses will include descriptive and comparative statistics to summarize and explore patterns in process and outcome domains across measurement points and settings. Descriptive analyses will be used to explore intervention delivery (eg, coverage, frequency and dose, duration, and adaptations), while comparative analyses will be applied to examine associations and potential relationships between process variables, contextual factors, and short- and long-term outcomes. Depending on data type, distributions, and study-specific requirements, appropriate parametric and nonparametric tests will be conducted using established statistical software, such as R (R Foundation for Statistical Computing), IBM SPSS, or equivalent.

#### Qualitative Data

For all qualitative data (Tables 1-6), an inductive-deductive approach will be applied guided by a thematic analysis outlined by Braun and Clarke [40], using NVivo software. Interviews will be audio-recorded and transcribed verbatim. Data analysis will follow an iterative process, allowing insights from initial telephone interviews, surveys, nonparticipant observations, and FGIs to inform subsequent data collection. The analysis process will follow a customary process, including repeated reading, coding, and categorizing, with gradually increasing abstraction. The final abstraction level will be determined based on the depth of data and the requirement of the different specific study aims.

#### Triangulation

In line with the convergent parallel mixed methods study design, all data will be analyzed in a stepwise manner. Initially, quantitative and qualitative data will be analyzed independently from each other, followed by iterative triangulation according to Creswell and Plano-Clark [41]. The breadth and complexity of the data, combined with data collection before, during, and after the intervention phase, means that different parts of the quantitative and qualitative results will be merged in multiple combinations to meet each specific aim. The formulation of the study aims reflects different analytic logics (convergent, exploratory, and explanatory) across aims, which will guide the integration of quantitative and qualitative findings, without altering the overall convergent design. Based on each aim, integration will occur through systematic comparison and complementary quantitative and qualitative results to inform understanding of observed outcomes. The different combinations of quantitative and qualitative data used in triangulation are outlined in Tables 1-6 for each process domain. Integration will be achieved through merging quantitative and qualitative results using complementary strategies, including joint displays, side-by-side comparisons, narrative weaving by aim, and matrix mapping of integrated findings to components of the underlying logic model, following the typology described by Fetters et al [42]. The logic model will further serve as a theoretical framework to guide the interpretation of interrelationships between contextual factors, implementation processes, mechanisms of impact, and intervention outputs (Figure 1). This strategy aims to strengthen the trustworthiness [43] of the process evaluation and to evaluate and refine the underlying logic model in alignment with the empirical results.

### Ethical Considerations

The project was approved by the Swedish Ethics Review Authority (IDs 2023-02402-01 and 2024-01407-02). Informed consent was collected from the participants before data collection. Project datasets will be stored in secure storage for research data provided by Region Västmanland. Members of the Systematic and Integrated Occupational Health and Patient Safety (SIOHPS) project core team will be given access to the cleaned datasets. To ensure confidentiality, all data forms and data files will be pseudonymized, and project team members will be blinded to any identifying participant information. The code key will be stored separately. Access to the code key will be restricted to researchers in the core research team.

## Results

Research funding for the project began in January 2023. The development phase was completed in the beginning of 2024, including refinement of the program theory, intervention components, and evaluation design in close collaboration with key stakeholders. The evaluation phase started in June 2024, with completion planned in early February 2026. The effectiveness trial protocol has been published, and description of the development phase is currently in preparation. Quantitative data collection for two of three clusters (baseline, 4- and 8-month follow-up) is complete, and data cleaning of these datasets is in progress. Interim feedback has been provided to participating units in clusters I and II. All qualitative material collected to date (contextual mapping and FGIs) has been fully transcribed. Planning for the final data collection for cluster III is underway and scheduled for the end of January and early February 2026, including the 8-month follow-up survey and FGIs. Data analysis will start in February-March 2026. Results will be disseminated through publications and conference presentations starting in 2026.

## Discussion

### Summary

This protocol outlines the process evaluation embedded in the SIOHPS trial, an effectiveness-implementation hybrid type 1 study assessing an integrated approach to systematic OSH and PS management. Conducting a process evaluation alongside the trial is essential to clarify how the intervention is delivered, how contextual factors influence implementation, and how through them change is generated [18,44]. Because the hybrid type 1 design primarily targets effectiveness [22], fidelity focuses on intervention fidelity, including fidelity of delivery and participant adherence [36] instead of implementation fidelity. The logic model (Figure 1) will guide the analytical process [18] and support examination of how fidelity, adaptations, and contextual conditions interact across system levels.

A mixed methods design [41] is appropriate for capturing the complexity of the SIOHPS intervention. Quantitative process data collected at multiple time points will describe implementation patterns and indicators of sustainability, while qualitative data will be gathered iteratively throughout implementation. This reflects the intervention’s complexity and the dynamic nature of its implementation context. Multiple mixed methods will support meaningful data integration and accommodate emerging contextual changes. Coproduction with stakeholders will help ensure that diverse perspectives are presented.

### Comparison With Prior Work

Implementation of complex interventions often occurs in organizational environments characterized by imbalances between job demands and available resources [23]. Consistent with previous research, factors such as organizational readiness [45], leadership engagement [46], and traditional separation of systematic OSH management and PS work are likely to influence the implementation of SIOHPS.

Organizational interventions aimed at strengthening PS culture have shown positive effects on HCW working conditions and health [47], yet few are designed to support systematic OSH management or reinforce OSH culture. Despite the interdependence between OSH and PS, a recent scoping review did not identify interventions targeting improvements in both safety cultures simultaneously (M Lohela-Karlsson, Associate Professor, unpublished data, January 2026). This gap likely reflects longstanding organizational silos within health care. Within SIOHPS, the work processes are integrated, which is essential for influencing both PS culture and OSH culture, thereby supporting the interventions primary long-term outcomes. Such organizational integration introduces additional complexity, as the intervention interacts dynamically with local structures, workflows, and contextual conditions. This aligns with contemporary perspectives emphasizing the need to embrace complexity in the design and evaluation of organizational interventions [48], reinforcing the importance of a rigorous process evaluation.

Balancing fidelity and adaptation [49] are particularly relevant in complex interventions. Achieving fidelity, while allowing contextually appropriate adaptations, requires preserving core components while tailoring implementation to local needs. This process evaluation will examine how this balance is negotiated across units and regions. Although specific implementation strategies are not tested, mapping contextual factors at micro and meso levels, together with documentation of barriers and facilitators, will support a nuanced understanding of how SIOHPS is enacted in practice.

With the Safer Culture Framework, encompassing enabling factors and enacting behaviors, this evaluation adopts a theory-informed lens that strengthens understanding of integrated OSH and PS management interventions. This approach positions key factors for safety culture as the key unifying mechanism connecting the two fields and influences how integrated interventions produce change. The SIOHPS evaluation therefore offers a theoretically grounded example of how mechanisms of impact operate across organizational, group, and individual levels, thereby advancing the understanding of mechanisms of change [50].

### Strengths and Limitations

A strength of this process evaluation is the use of a logic model to guide data collection and analysis. This facilitates systematic assessment of fidelity, adaptations, contextual conditions, and mechanisms of impact and supports iterative refinement of theoretical assumptions. The mixed methods design and longitudinal data collection allow for the examination of temporal patterns and triangulation of findings across diverse data sources. Coproduction provides valuable insights into multiple perspectives on the implementation process.

The process evaluation also has potential limitations. Achieving adequate response rates in web-based surveys remains challenging [51] and will require close monitoring and active involvement of first-line managers and internal facilitators. The round-the-clock operational context of participating units might limit opportunities to convene support functions and internal facilitators for focus groups. The complexity of the SIOHPS intervention and the substantial volume of data collected also present analytical challenges. To mitigate these issues, analytic procedures will be anchored in the logic model and supported by iterative triangulation across methods.

### Conclusion

This process evaluation is designed to illuminate how SIOHPS is implemented across diverse real-world settings and to identify the mechanisms through which it generates both short- and long-term outcomes. Through contextual mapping, exploration of barriers and facilitators, and examination of fidelity and adaptations, the evaluation is expected to generate insights that can inform future refinements of the SIOHPS intervention and guide the selection of appropriate implementation strategies for broader scale-up. The mixed methods approach, combined with iterative engagement with stakeholders, will enhance the robustness and transferability of findings and contribute to advancing knowledge on integrated complex organizational interventions, such as OSH and PS interventions.

## Appendix

Multimedia Appendix 1 Peer-review report from the Swedish Research Council for Health, Working Life and Welfare (FORTE).

## Abbreviations

FGI: focus group interview
HCW: health care worker
MRC: Medical Research Council
OSH: occupational safety and health
PI: principal investigator
PS: patient safety
SIOHPS: Systematic and Integrated Occupational Safety and Health and Patient Safety Management Systems

## Data Availability

The Systematic and Integrated Occupational Safety and Health and Patient Safety Management Systems (SIOHPS) project group will be open for collaboration with other researchers, and data will be shared, provided that a research plan is submitted to the project group and aligns with the ethical approval.
