# Supplementary material for: Process Evaluation of a Structured Method for Systematic and Integrated Occupational Safety and Health and Patient Safety Management Systems (SIOHPS): Protocol for a Convergent Parallel Mixed Methods Study
Source: JMIR Res Protoc. 2026 Jan 27;15:e89185. doi: 10.2196/89185 (PMC12892028; doi:10.2196/89185)
Supplement: Multimedia Appendix 1 [file resprot_v15i1e89185_app1.pdf]

FORTE: Forskningsrådet för hälsa, arbetsliv och välfärd

Beredningsgruppens yttrande

2022-00404	Malin Lohela Karlsson	Beredningsgrupp: AH 2022

**Utlysningsnamn:** Forskningsprojekt 2022	**Bidragsform:** Projekt
**Projekttitel (svenska):** SAMSA: Ett verktyg för förbättrad hälsa, vårdkvalitet genom integrering av arbetsmiljö- och patientsäkerhetsarbete i vardagen.	**Sökt inriktning:** Fritt

Bedömning

Vetenskaplig kvalitet

Research aim and questions are relevant for research. The project has a well described and high-quality design. The researchers are recommended to pay particular attention to integration of intervention outcomes in daily operations.

Genomförbarhet

Realistic plan for implementation. Principal investigator and other researchers are qualified and have the relevant competences. The budget is realistic.

Relevans

Both safety and health of employees and patient safety are relevant and challenging issues in hospitals. The integration of the two issues gives a larger possibility for a relevant impact. Relevant and realistic plans for application and dissemination.

Sammanvägd bedömning

A qualified project both scientifically and societally relevant.

Förslag till beslut (bevilja, reserv, avslå)

Approve
